# Abnormal anxiety- and depression-like behaviors in mice lacking both central serotonergic neurons and pancreatic islet cells

**DOI:** 10.3389/fnbeh.2014.00325

**Published:** 2014-09-23

**Authors:** Yun-Fang Jia, Ning-Ning Song, Rong-Rong Mao, Jin-Nan Li, Qiong Zhang, Ying Huang, Lei Zhang, Hui-Li Han, Yu-Qiang Ding, Lin Xu

**Affiliations:** ^1^Key Laboratory of Animal Models and Human Disease Mechanisms of Chinese Academy of Sciences and Yunnan Province, and KIZ/CUHK Joint Laboratory of Bioresources and Molecular Research in Common Disease, and Laboratory of Learning and Memory, Kunming Institute of Zoology, Chinese Academy of SciencesKunming, China; ^2^Kunming College of Life Science, University of Chinese Academy of SciencesBeijing, China; ^3^Key Laboratory of Arrhythmias, Ministry of Education of China, East Hospital, Tongji University School of MedicineShanghai, China; ^4^Department of Anatomy and Neurobiology, Tongji University School of MedicineShanghai, China

**Keywords:** diabetes mellitus, anxiety, depression, serotonin, Pet1, adult neurogenesis

## Abstract

Dysfunction of central serotonin (5-HT) system has been proposed to be one of the underlying mechanisms for anxiety and depression, and the association of diabetes mellitus and psychiatric disorders has been noticed by the high prevalence of anxiety/depression in patients with diabetes mellitus. This promoted us to examine these behaviors in central 5-HT-deficient mice and those also suffering with diabetes mellitus. Mice lacking either 5-HT or central serotonergic neurons were generated by conditional deletion of *Tph2* or* Lmx1b* respectively. Simultaneous depletion of both central serotonergic neurons and pancreatic islet cells was achieved by administration of diphtheria toxin (DT) in *Pet1*-Cre;Rosa26-DT receptor (DTR) mice. The central 5-HT-deficient mice showed reduced anxiety-like behaviors as they spent more time in and entered more often into the light box in the light/dark box test compared with controls; similar results were observed in the elevated plus maze test. However, they displayed no differences in the immobility time of the forced swimming and tail suspension tests suggesting normal depression-like behaviors in central 5-HT-deficient mice. As expected, DT-treated *Pet1*-Cre;Rosa26-DTR mice lacking both central serotonergic neurons and pancreatic islet endocrine cells exhibited several classic diabetic symptoms. Interestingly, they displayed increased anxiety-like behaviors but reduced immobility time in the forced swimming and tail suspension tests. Furthermore, the hippocampal neurogenesis was dramatically enhanced in these mice. These results suggest that the deficiency of central 5-HT may not be sufficient to induce anxiety/depression-like behaviors in mice, and the enhanced hippocampal neurogenesis may contribute to the altered depression-like behaviors in the 5-HT-deficient mice with diabetes. Our current investigation provides understanding the relationship between diabetes mellitus and psychiatric disorders.

## Introduction

The central serotonin (5-HT) system is known to be involved in emotion, learning and memory (Barnes and Sharp, 1999; Duman and Voleti, 2012), and 5-HT deficiency in the brain is believed to be a major causative factor in anxiety and depression (Brigitta, 2002; Leonardo and Hen, 2006). Selective 5-HT reuptake inhibitors (SSRIs), which can promote monoamine system functions, attenuate anxiety-like behaviors (Graeff et al., 1996) and also produce certain beneficial effects in patients with depression (Artigas et al., 1996; Blier and Ward, 2003). They are currently the most commonly prescribed class of antidepressants (Santarelli et al., 2003), although the exact molecular mechanisms behind their antidepressant effects are not fully understood. The use of SSRIs may help to alleviate the symptoms of depression by promoting adult neurogenesis in the hippocampus (Malberg et al., 2000; Santarelli et al., 2003).

The global prevalence of diabetes mellitus has been steadily increasing over the past few decades (Alberti et al., 1998; Wild et al., 2004). The diabetes type I is caused by the destruction of pancreatic islet endocrine cells (Butler et al., 2003), whose normal function is to secrete hormones that regulate blood glucose homeostasis, such as insulin (Lenoir et al., 2011). The typical symptoms of diabetes include polyuria, polyphagia and polydipsia as well as the elevation of blood glucose level (Alberti et al., 1998; Cooke and Plotnick, 2008). Despite the symptoms of diabetes mellitus and psychiatric disorders (e.g., anxiety and depression) are very much non-overlapping, these two disorders are somehow co-occurred (Ciechanowski et al., 2000; Anderson et al., 2001). For example, the prevalence of anxiety/depression is 2–3 times higher in patients with diabetes mellitus compared with the general population (Popkin et al., 1988; Blanz et al., 1993), and this observation has sparked the hypothesis that an increased risk of developing anxiety and depression may be a complication of diabetes mellitus. Some clinical studies have also shown that anxiety/depression may be a risk factor for diabetic complications as well as increased morbidity and mortality from diabetes mellitus (Anderson et al., 2001).

Our previous studies have reported that transcription factor *Lmx1b* is required for the development of central serotonergic neuron (Ding et al., 2003), and conditional deletion of this gene results in the loss of serotonergic neurons therefore offering a central 5-HT-deficient mouse model (Dai et al., 2008). Tryptophan hydroxylase (Tph2) is the key enzyme for 5-HT synthesis in the brain (Zhang et al., 2004), and specific deletion of *Tph2* leads to the central 5-HT deficiency without affecting the survival of the serotonergic neurons (Savelieva et al., 2008; Kriegebaum et al., 2010). The prototypical serotonergic transcription factor *Pet1* is expressed in both central serotonergic neurons and pancreatic islet cells (Hendricks et al., 1999, 2003; Ohta et al., 2011), and *Pet1*-driven Cre expression was used to simultaneously deplete these two kinds of cells in *Pet1*-Cre;Rosa26-diphtheria toxin receptor (DTR) mice in adulthood. The aim of the present study was to examine whether central 5-HT deficiency results in abnormal anxiety- and depression-like behaviors, and to explore the possible involvement of diabetes mellitus in these behaviors and the underlying mechanisms.

## Materials and methods

### Mice

Mice lacking central serotonergic neurons (*Pet1*-Cre; *Lmx1b*^flox/flox^) were generated by crossing *Pet1*-Cre mice to flox *Lmx1b* mice (hereafter referred to as PC/Lmx1b) as reported in our previous study (Dai et al., 2008). The other central 5-HT-deficient mice (*Pet1*-Cre;*Tph2*^flox/flox^; referred to as PC/Tph2) used in this study were obtained by crossing *Pet1*-Cre mice to flox *Tph2* mice as described in our previous report also, which contained central serotonergic neurons but failed to synthesize 5-HT (Kriegebaum et al., 2010). To simultaneously deplete both the central serotonergic neurons and pancreatic islet cells, *Pet1*-Cre;Rosa26-DTR (referred to as PC/DTR) mice were generated by crossing Rosa26-DTR mice (Buch et al., 2005) to *Pet1*-Cre mice. In addition, *Pet1*-Cre/Rosa26-LacZ mice were generated by crossing Rosa26-LacZ mice (Soriano, 1999) to *Pet1*-Cre mice; this was used to examine the Cre activity in the pancreas and brain. Furthermore, *Pomc*-GFP mice (Overstreet et al., 2004) were crossed with PC/DTR mice to obtain PC/DTR/*Pomc*-GFP mice, which were used to visualize immature neurons in the subgranular zone (SGZ) of the dentate gyrus.

In the set of experiment for depleting central serotonergic neurons and pancreatic islet cells, DT was intraperitoneally injected once daily for 2 days (20 ng/g body weight; Sigma) in PC/DTR or PC/DTR/Pomc-GFP mice and control mice. Behavioral tests and immunostaining analysis were performed 3–4 weeks after DT injection. Animals were group-housed (4–5 mice per cage) in a thermo-regulated environment (22–24°C) with *ad libitum* access to water and food, and on a 12-h light/dark cycle. All animal care and experimental protocols were approved by the Animal Research Committees of the Kunming Institute of Zoology, Chinese Academy of Sciences and the Tongji University School of Medicine, China.

### X-gal staining

X-gal staining was used to detect Cre recombinase activity in *Pet1*-Cre mice. Two-week old *Pet1*-Cre;Rosa26 mice were deeply anesthetized, then their pancreas and brain were dissected in cold 0.01 M phosphate buffered saline (PBS; pH 7.4), fixed in 4% paraformaldehyde in PBS for 2 h at 4°C, rinsed briefly in PBS and cryoprotected with 30% sucrose in PBS overnight. Twenty-μm thick sections were then cut with a cryostat. The sections were stained with X-gal solution containing 1 mg/ml X-gal, 2 mM MgCl_2_, 5 mM K_3_Fe(CN)_6_ and 5 mM K_4_Fe(CN)_6_ for 10–16 h at 37°C, and then slightly counterstained with neutral red.

### Immunohistochemistry, bromodeoxyuridine (BrdU) labeling and *in situ* hybridization

After perfusing with 4% paraformaldehyde, brains were removed and post-fixed overnight. Forty-μm brain sections were cut and incubated with mouse anti-bromodeoxyuridine (BrdU) (1:300; Calbiochem) or goat anti-NeuroD (1:400; Santa Cruz) primary antibody at 4°C overnight, followed by incubation with the appropriate biotinylated secondary antibody (1:500; Vector) for 3 h and Cy3-conjugated streptavidin (1:1000; Jackson ImmunoResearch) for 1 h at room temperature. For BrdU pulse labeling, mice were injected with BrdU (50 mg/kg body weight) four times at 2-h intervals, and then sacrificed for analysis 2 h after the last injection. Prepared sections were treated with 0.01 M citrate buffer at 95°C for 5 min, then incubated in 2 N HCl at 37°C for 20 min and in 0.1 M sodium borate for 10 min, and finally washed in PBS. The treated sections were then immunostained with mouse anti-BrdU antibody as described above. *In situ* hybridization for detection of *Pet1* mRNA expression was performed as reported in our previous study (Song et al., 2011). Images were captured using a Nikon epifluorescence microscope (Eclipse 80i) or a confocal microscope (TCS SP5, Leica). Cell counts in the dorsal raphe nucleus and hippocampal SGZ were performed in every sixth section from each animal. As the factor of volumes of the counted brain regions were not included, our counting method may lead to an overestimation or underestimation of the real number of serotonergic neurons in the raphe nucleus and new-born neurons in the dentate gyrus.

### Diabetes related examination

Body weight was measured manually at the beginning of all experiments. Blood glucose level was measured from the mice tail vein blood using the glucometer (ONE TOUCH Ultra Glucometer, Johnson & Johnson Medical [China] CO., Ltd.) after 3-h fasting. The quantities of food and water intake were assessed by a metabolic cage and the monitoring software (Panlab Harvard). Diptheria toxin injected control mice were individually placed in one cage and allowed free access to food and water during a 24-h period, and the quantities were recorded at each hour; DT-injected PC/DTR mice were treated in the same way. The data of food and water intake were recorded by the monitoring software.

### Behavioral observation

Adult (3–5 months old) male mice were used in the following behavioral observation. Physiological cycle in adult female mice may cause the instability of some hormones (e.g., estrogen), and this may influence the behavioral performances and therefore complicate dissecting the relationship between the central 5-HT deficiency and behavioral studies. The experiments were performed during 9:00 am to 17:00 pm, and animal behaviors were videotaped. It should be noted that the behavioral data acquisition and analysis were performed by a double-blind manner; people performed these were blind to genotypes. Animals were habituated in the test room for 2 h before starting the experiments.

In this study, multiple mouse lines were used, and they were generated by crossing two lines of mice thus having a mixed genetic background. Behavioral performances are different in different mouse stains, and the genetic background of control mice was well controlled in the following ways. First, to minimize the contribution of strain differences, the mice lines used in this study have been intercrossed by sister-brother mating over seven generations to get a congenetic background. Second, in each set of behavioral observations, comparison was made only between gene-modified mice (i.e., PC/Lmx1b, PC/Tph2 and PC/DTR) and wild type mice (controls) from the same litters, which should have the same mixed genetic background.

### Light/dark box test

We used a light/dark box (ENV-510 test environment) that were divided into two equally sized compartments (13.5 × 27 × 20.3 cm) together with the activity monitoring software (Activity Monitor; Med Associates) to evaluate the anxiety levels. Mice were placed in the central light box facing the dark box and were allowed to freely explore for 5 min. The amount of time spent in the light box and the frequency of transitions between the light and dark boxes were recorded. The data of PC/Tph2 mice were recorded by an experienced observer by means of a video/computer system, while the data of PC/DTR mice were measured by the activity monitoring software (Med Associates).

### Elevated plus maze

Elevated plus maze (Med Associates) also was used to test anxiety-like behaviors (Lister, 1987; Weisstaub et al., 2006), and the platform was elevated 74 cm above the floor, which consisted of two open (35 × 6 cm) and two closed (35 × 6 × 22 cm) arms and a connecting central zone (6 × 6 cm). Mice were placed on the central platform facing an open arm and were allowed to freely explore the maze for 5 min. The amount of time spent in the open arms and the frequency of transitions between the open and closed arms were recorded. The data of PC/Tph2 mice were measured by an experienced observer who was blind to genotypes by means of a video/computer system, while the data of PC/DTR mice were recorded by the monitoring software.

### Forced swimming test

This test was used to assess behavioral despair in mice, which is often used to approximate some aspects of depression in humans (Porsolt, 2000; Nestler et al., 2002) as described previously (Porsolt et al., 1977; David et al., 2003; Tian et al., 2011). The apparatus consisted of a glass cylinder (24 cm height, 16 cm diameter) that was filled with water to a depth 18 cm and maintained at 25 ± 1°C. Mice were forced to swim 6 min, and the time spent immobile during the last 4 min was recorded by an experienced observer who was blind to genotype. They were considered immobile when they stopped struggling, only moved slightly and occasionally to keep their nose above the water surface.

### Tail suspension test

Time spent immobile in this test was also used to measure behavioral despair (Steru et al., 1985; Castagne et al., 2011). Mice were suspended by the tail from a vertical stainless steel hook (distance from floor: 20 cm) using adhesive tape (distance from tip of tail: 2 cm). The immobility time was recorded by the activity monitoring software (Med Associates, Tail suspension system) during suspended the mice 6 min. They were considered immobile when they hung passively without any movement.

### Open field test

The ENV-510 test environment equipped with infrared beams and Activity Monitor (Med Associates) were used to evaluate motor activity in the open field test. Mice were placed in a Plexiglas box (27 × 27 × 20.3 cm) to freely explore the chamber for the duration of the test session and the locomotor activity was monitored over a 30-min period, and the data were recorded by each beam break as one unit of exploratory activity using the activity monitoring software (Med Associates).

### Rotarod test

Mice were placed on the rotating rod and the latency to fall was measured with a 4–40 rpm accelerating speed during 5 min (Panlab Harvard). Each mouse was tested three times at 1-h intervals after habituating 2 min each time, and was placed on the rod for a maximum of 15 min per trial.

### Statistical analysis

All data were expressed as mean ± SEM. Comparison between two groups was conducted by unpaired Student’s *t*-test, the food and water intake tests were analyzed by using a repeated measure ANOVA followed by the Tukey Honestly Significantly Different (HSD) test. Differences were considered statistically significant if *p* < 0.05.

## Results

### Abnormal behaviors in the central 5-HT-deficient mice

**Figure 1:**
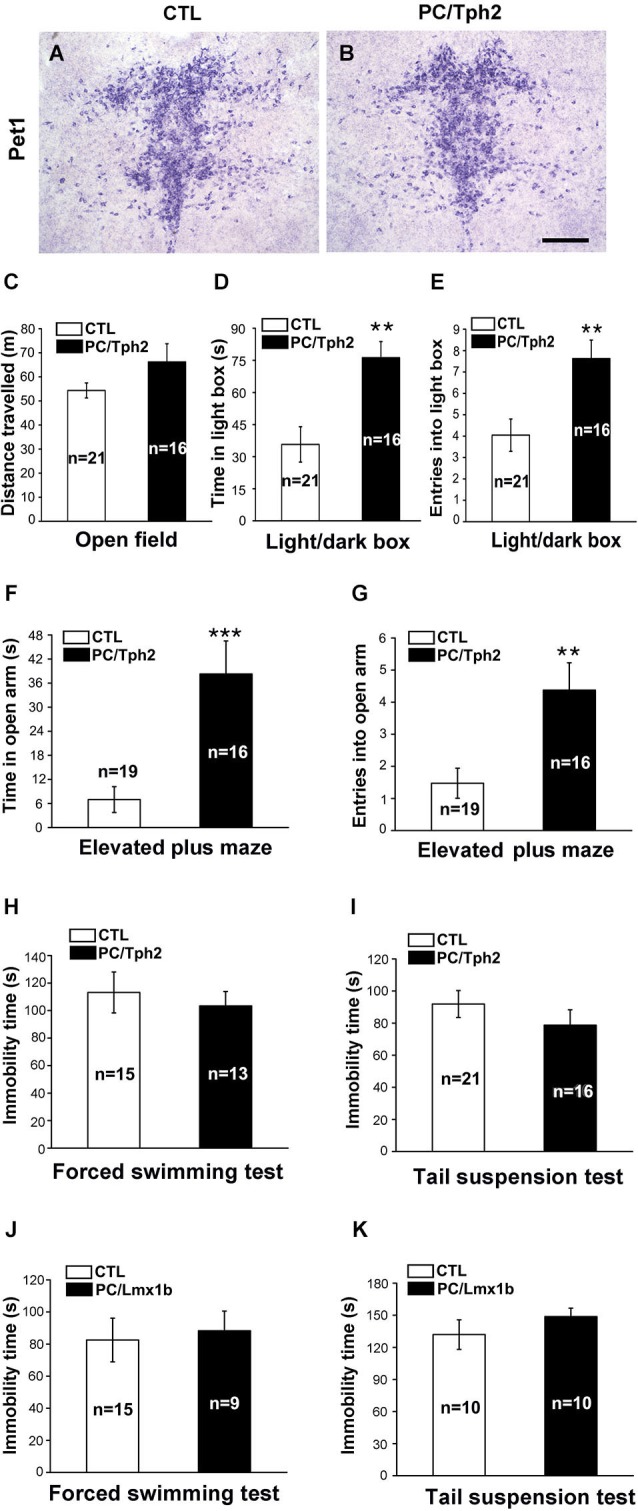
**Decreased anxiety-like behaviors and unchanged depression-like behaviors in the central 5-HT-deficient mice. (A, B)** Pet1-expressing cells still exist in the dorsal raphe nucleus of PC/Tph2 mice. Scale bar: 300 μm. **(C)** There are no differences between PC/Tph2 mice and controls in the total distance traveled in the open field test during a 30-min period. **(D, E)** In the light/dark box test, PC/Tph2 mice spend more time in the light box (**D**, *p* < 0.01), and enter more times into the light box compared with the control mice (**E**, *p* < 0.01). **(F, G)** In the elevated plus maze test, longer time in the open arms is spent by PC/Tph2 mice (**F**,*p* < 0.001), and they also enter more times into the open arms compared with controls (**G**, *p* < 0.01). **(H)** Immobility time in the forced swimming test shows no differences between PC/Tph2 mice and control mice. **(I)** Immobility time in the tail suspension test is not different between PC/Tph2 mice and control mice. **(J)** Immobility time in the forced swimming test shows no differences between PC/Lmx1b mice and control mice. **(K)** Immobility time in the tail suspension test shows no differences between PC/Lmx1b mice and control mice. “n” denotes the animal number in figures. CTL: control. All data are expressed as mean ± SEM. ** and *** indicate *p* < 0.01 and *p* < 0.001.

We first detected the anxiety- and depression-like behaviors in PC/Tph2 mice (*Pet1*-Cre;*Tph2*^flox/flox^) in which *Tph2*, the key enzyme for 5-HT synthesis, was deleted in the central serotonergic neurons as reported previously (Kriegebaum et al., 2010). Inactivation of *Tph2* did not affect the survival of serotonergic neurons in the brain as revealed by the expression of *Pet1* in PC/Tph2 mice (Figures 1A,B). The spontaneous locomotor activity shown by the travelled distance in the open field was not different between the PC/Tph2 and control mice (Figure 1C). In the light/dark box test, PC/Tph2 mice displayed more time in the light box (Figure 1D), and more times of entries into the light box compared with controls (Figure 1E). Similar results were obtained in the elevated plus maze test, shown by spending more time in the open arms (Figure 1F) and more times of entries into the open arms in PC/Tph2 mice compared with controls (Figure 1G). On the other hand, in the forced swimming (Figure 1H) and tail suspension tests (Figure 1I), no significant differences were detected in the immobility time between the PC/Tph2 and control mice. These results suggest that the level of anxiety-like behaviors may be decreased but that of depression-like behaviors is likely unchanged in PC/Tph2 mice.

The unchanged depression-like and decreased anxiety-like behaviors in PC/Tph2 mice are somehow inconsistent with the monoamine deficiency hypothesis of depression which is supported by the anti-depressive effect of SSRIs. To further confirm this, we set out to examine these behaviors in PC/Lmx1b (*Pet1*-Cre;*Lmx1b*^flox/flox^) mice lacking the central serotonergic neurons (Dai et al., 2008). Reduced anxiety-like behaviors revealed by both the elevated plus maze and novelty suppressed feeding tests has been published in our previous study (Dai et al., 2008). Here, we found that in the forced swimming and tail suspension tests, PC/Lmx1b mice behaved similarly as PC/Tph2 mice; the immobility time was not different from control mice (Figures 1J,K). Taken together, these results suggest the possibility that 5-HT deficiency in the brain may not be sufficient to alter the depression-like behaviors but may lead to a reduced anxiety level in mice.

### Pet1-expressing cells are located in both pancreatic islet and raphe nuclei

Previous report has indicated that *Pet1* is expressed in the pancreatic islets and shows a restricted expression to the central serotonergic neurons in the brain (Hendricks et al., 1999, 2003; Ohta et al., 2011). We crossed Rosa26-DTR mice with *Pet1*-Cre mice to generate the *Pet1*-Cre;Rosa26-DTR (PC/DTR) mice, which led to deplete the Pet1-expressing cells: pancreatic islet cells and central serotonergic neurons in adulthood. The PC/DTR mice have the DTR sequence inserted into the ROSA26 locus, downstream of a loxP-flanked STOP cassette (Buch et al., 2005). Excision of the STOP cassette through Cre recombinase activity therefore renders these cells susceptible to DT-mediated ablation.

**Figure 2:**
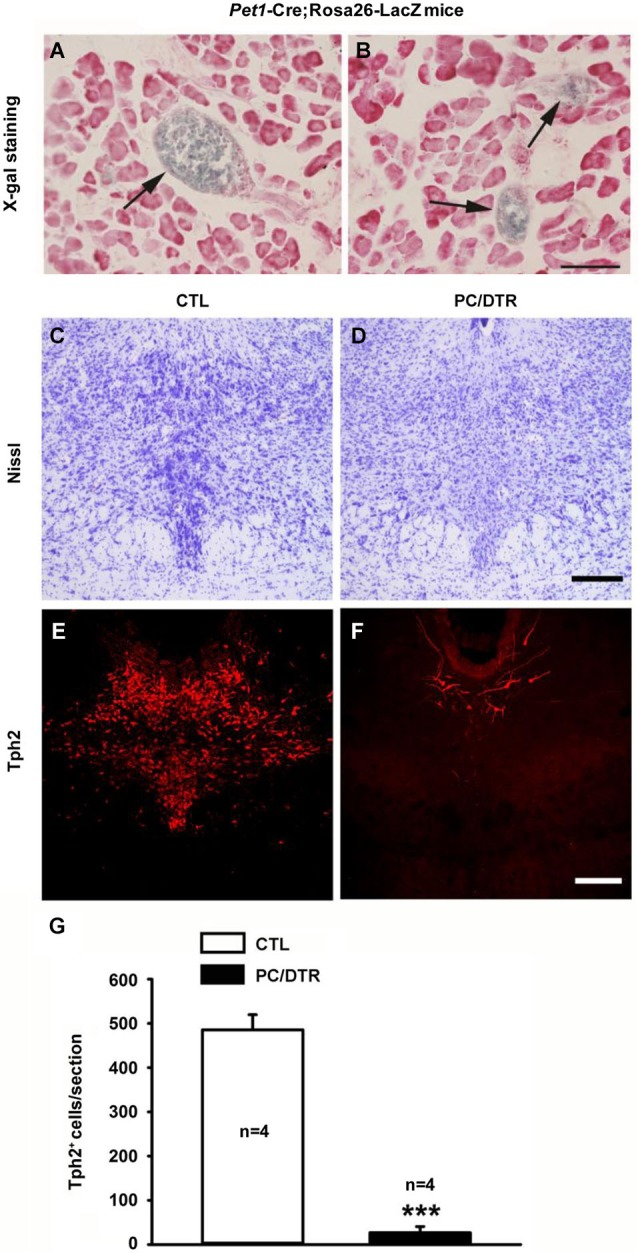
**Pet1-expressing cells are located in pancreatic islet of** ***Pet1*-Cre;Rosa26-LacZ mice and raphe nuclei of DT-treated PC/DTR mice**. **(A, B)** X-gal staining reveals that Cre recombinase is specifically expressed in the islet of the pancreas (arrows) of *Pet1*-Cre/Rosa26-LacZ mice. Sections are counterstained with Neutral red. Scale bar: 90 μm. **(C, D)** Nissl staining shows intensely-stained serotonergic neurons (arrowheads) in the dorsal raphe nucleus of control mice (**C**), but not in DT-treated PC/DTR mice (**D**). **(E, F)** Immunohistochemical staining shows that nearly all *Tph2*-positive cells are lost from the dorsal raphe nucleus of DT-treated PC/DTR mice (**F**) compared with controls (**E**) (*p* < 0.01). **(G)** Quantitative analysis of the results shown in **(E)** and **(F)**. CTL: control. All data are expressed as mean ± SEM. *** *p* < 0.001.

We first confirmed the functional Cre recombinase by crossing *Pet1*-Cre mice with Rosa26-LacZ reporter mice (Soriano, 1999) and assessing β-gal activity. In the peripheral system of *Pet1*-Cre;Rosa26-LacZ mice, X-gal-positive staining was only observed in the islets of the pancreas (Figures 2A,B). In our previously published observations (Dai et al., 2008), X-gal staining was detected selectively in the central serotonergic neurons of the raphe nuclei in the brain. We next looked at the expression of *Tph2*, a specific central serotonergic neuron marker in the brain stem. Along with Nissl staining (Figures 2C,D), *Tph2* immunostaining revealed that the central serotonergic neurons were largely depleted in the raphe nuclei of DT-treated PC/DTR mice compared with controls (Figures 2E–G). Our results here indicate that both the pancreatic islet cells and central serotonergic neurons could be depleted by the systemic administration of DT in adult PC/DTR mice.

### DT-treated PC/DTR mice exhibit typical diabetes mellitus-associated symptoms

**Figure 3:**
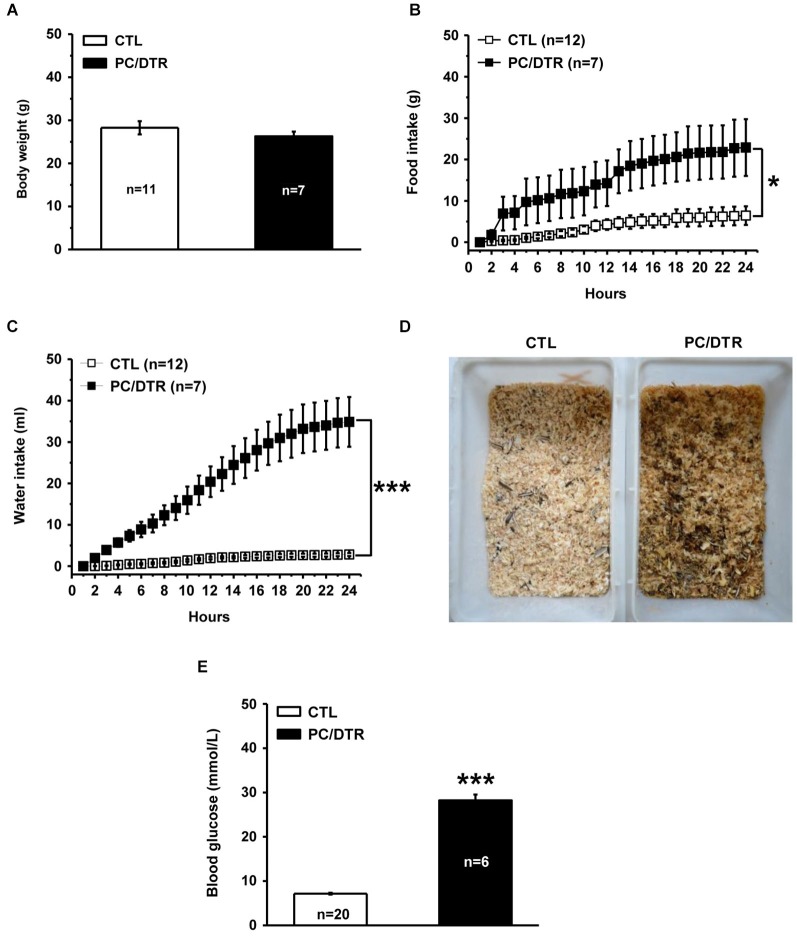
**DT-treated PC/DTR mice exhibit typical diabetic symptoms**. **(A)** Average body weight in DT-treated PC/DTR mice is comparable to that of control mice. **(B, C)** Cumulative food (**B**, *F*_(1,17)_ = 7.007, *p* < 0.05) and water (**C**, *F*_(1,17)_ = 46.184, *p* < 0.001) intake levels over a 24-h period are significantly higher in DT-treated PC/DTR mice than in controls. **(D)** DT-treated PC/DTR mice urinate much more than control mice, as revealed by the overall appearance of their litter. **(E)** Blood glucose levels are markedly increased in DT-treated PC/DTR mice compared with controls (*p* < 0.01). CTL: control. All data are expressed as mean ± SEM. * *p* < 0.05, *** *p* < 0.001.

Functional or physical damage of pancreatic islet cells can result in insulin deficiency, and in turn lead to the onset of diabetes mellitus (DeFronzo et al., 1992). Therefore, we next determined whether selective ablation of islet cells gave rise to diabetic symptoms in the DT-treated PC/DTR mice. We found that despite these mice showed normal body weight (Figure 3A), they exhibited increased food and water intake compared with controls (Figures 3B,C), which are reminiscent of two classic diabetic symptoms: polyphagia and polydipsia. In addition, the litter in the DT-treated PC/DTR mice home cages showed obvious signs of increased urination (Figure 3D). Importantly, blood glucose level after a 3-h fast was profoundly increased in the DT-treated PC/DTR mice compared with controls (Figure 3E). These results demonstrate that DT-mediated destruction of pancreatic islet cells leads to typical diabetic symptoms in adult PC/DTR mice.

### Altered anxiety- and depression-like behaviors in DT-treated PC/DTR mice

As mentioned previously, diabetes mellitus has been suggested to be an increased risk of developing anxiety and depression. Having observed typical symptoms of diabetes mellitus in our DT-treated PC/DTR mice, which also lacked the central serotonergic neurons, we hypothesized that anxiety- and depression-like behaviors would be changed in these mice.

**Figure 4:**
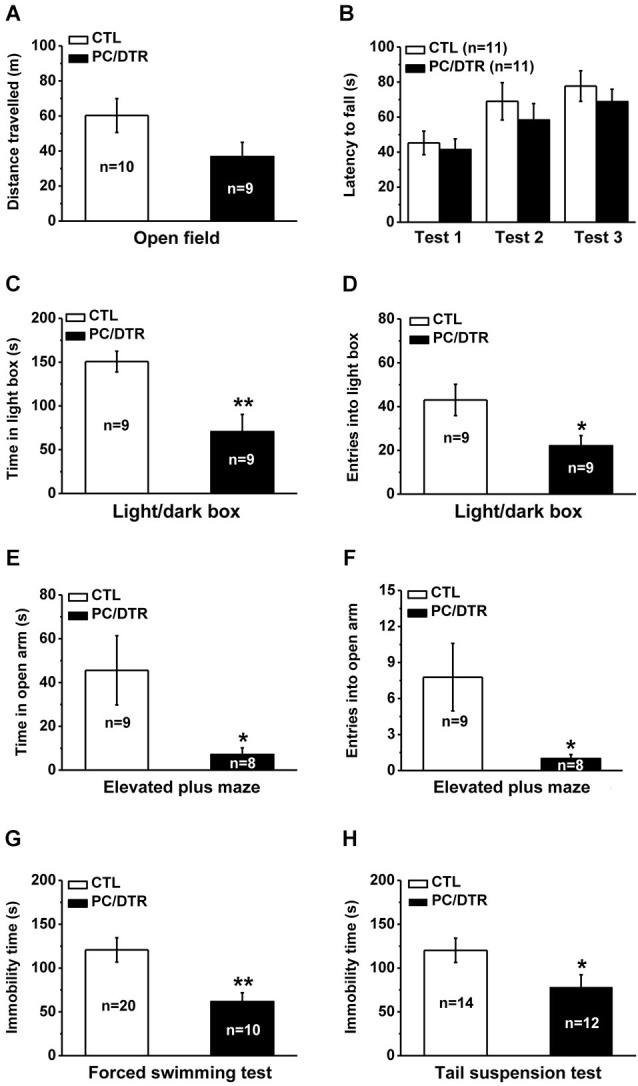
**Increased anxiety- but lowered depression-like behaviors in DT-treated PC/DTR mice**. **(A)** Total traveled distance during a 30-min period is comparable between DT-treated PC/DTR mice and controls in the open field test. **(B)** The latency to fall in the rotarod test is not significantly different between the two groups. **(C, D)** In the light/dark box test, DT-treated PC/DTR mice spend less time in the light box (**C**, *p* < 0.01), and enter fewer times into the light box compared with the control mice (**D**, *p* < 0.05). **(E, F)** In the elevated plus maze test, DT-treated PC/DTR mice spend less time in the open arms (**E**, *p* < 0.05), and enter into them fewer times compared with controls (**F**, *p* < 0.05). **(G)** Immobility time in the forced swimming test is significantly decreased in the DT-treated PC/DTR mice compared with the controls (*p* < 0.01). **(H)** Immobility time in the tail suspension test is significantly reduced in DT-treated PC/DTR mice (*p* < 0.05). All data are expressed as mean ± SEM. * *p* < 0.05, ** *p* < 0.01.

We first used the open field test to assess the changes in locomotor activity, and found that total traveled distance was not significantly different between the DT-treated PC/DTR and control mice (Figure 4A). The rotarod test was used to evaluate balancing ability, and no a significant difference was detected in the latency to fall between the two groups in three trial tests (Figure 4B). These results show that overall locomotor performance is normal in DT-treated PC/DTR mice.

We used the light/dark box and elevated plus maze tests to evaluate anxiety levels in our DT-treated PC/DTR mice. In the light/dark box test, they spent less time in the light box (Figure 4C) and entered it fewer times compared with the controls (Figure 4D). In the elevated plus maze test, the DT-treated PC/DTR mice spent less time exploring the open arms (Figure 4E) and entered into the open arms fewer times compared with control mice (Figure 4F). These results suggest that the anxiety levels are increased in the DT-treated PC/DTR mice.

We next used the forced swimming and tail suspension tests to investigate depression-like behaviors in the PC/DTR mice. Unexpectedly, immobility time was significantly decreased in the forced swimming (Figure 4G) and tail suspension tests (Figure 4H) of DT-treated PC/DTR mice, suggesting altered depression-like behaviors. These behavioral phenotypes were not due to any locomotor defects, because the open field and rotarod test results were both comparable to controls.

### Increased adult hippocampal neurogenesis in DT-treated PC/DTR mice

**Figure 5:**
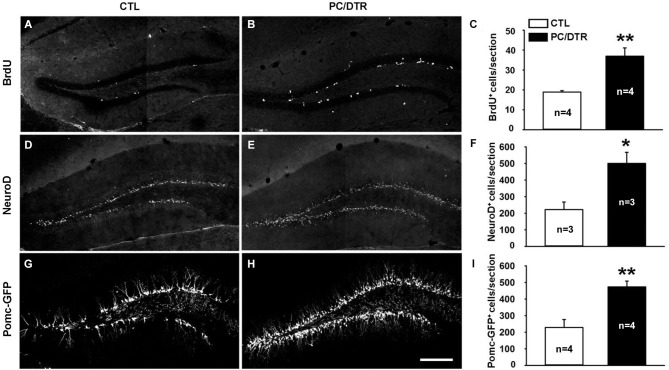
**Increased adult neurogenesis in the hippocampus of DT-treated PC/DTR mice**. **(A–F)** The numbers of BrdU-positive (**A–C**, *p* < 0.01) and NeuroD-positive cells (**D–F**, *p* < 0.05) are greatly increased in the SGZ of DT-treated PC/DTR mice compared with controls. **(G–I)** Green fluorescent protein-positive cells are also markedly increased in number in the SGZ of DT-treated PC/DTR/*Pomc*-GFP mice (*p* < 0.01). CTL: control. All data are expressed as mean ± SEM. * *p* < 0.05, ** *p* < 0.01.

Diabetic rodents induced by streptozotocin showed reduced adult hippocampal neurogenesis (Beauquis et al., 2008; Stranahan et al., 2008), while increased neurogenesis and cell proliferation rates were observed in db/db mice (Ramos-Rodriguez et al., 2014), which is a widely used genetic mouse model in diabetes research (Sharma et al., 2003). It has been shown that most of clinically used antidepressants (SSRIs being the most common) promote neurogenesis in the adult dentate gyrus, a characteristic that is thought to be critical for their antidepressant action (Malberg et al., 2000; Santarelli et al., 2003). Since our DT-treated PC/DTR mice exhibited both diabetic symptoms and anti-depressive behaviors, we detected whether adult hippocampal neurogenesis was changed in these mice, which might help to explain their behavioral phenotypes. We used BrdU to label the dividing cells and thereby assessed adult neural stem cell proliferation (van Praag et al., 1999; Kee et al., 2002; Taupin, 2007). Significantly more proliferating cells were observed in SGZ of the dentate gyrus in DT-treated PC/DTR mice compared with controls (Figures 5A–C). We also immunostained for the basic helix-loop-helix transcription factor NeuroD, a marker of differentiating newborn neurons (Lee et al., 1995; Miyata et al., 1999), and observed a pronounced increase in the number of NeuroD-positive cells in DT-treated PC/DTR mice compared with controls (Figures 5D–F). Immature neurons in the SGZ can be visualized also by GFP fluorescence in *Pomc*-GFP transgenic mice (Overstreet et al., 2004); as expected, more GFP-positive cells were observed in the SGZ of DT-treated PC/DTR/*Pomc*-GFP mice (Figures 5G–I). Taken together, these results demonstrate that the adult hippocampal neurogenesis is enhanced in DT-treated PC/DTR mice.

## Discussion

In the present study, we found that abnormal behaviors were observed in the two kinds of central 5-HT-deficient mice (PC/Tph2 and PC/Lmx1b). The data from the light/dark box and elevated plus maze tests suggest that the level of anxiety was decreased in these central 5-HT-deficient mice, but was increased in mice with both central 5-HT deficiency and diabetes (DT-treated PC/DTR mice), supporting the idea that the diabetes is a risk factor in developing anxiety. On the other hand, the unchanged immobility time revealed by the forced swimming and tail suspension tests in PC/Tph2 and PC/Lmx1b mice suggest that the central 5-HT deficiency may not be sufficient to induce depression-like behaviors in mice. In addition, we generated a novel transgenic mouse model in which Pet1-expressing cells were deleted in adulthood, and DT-treated PC/DTR mice exhibited typical symptoms of diabetes mellitus, as well as increased anxiety-like but lowered depression-like behaviors, to which the enhanced hippocampal neurogenesis may contribute. This mouse model (PC/DTR mice) may provide a novel insight to understand and investigate the underlying mechanisms of the clinical comorbidity between diabetes and psychiatric disorders.

Anxiety and depression are known to be associated with central 5-HT deficiency (Coppen, 1967; Naughton et al., 2000), which is supported by the facts that monoaminergic antidepressants attenuate anxiety-like behaviors (Graeff et al., 1996) and also produce beneficial effects in patients with depression (Artigas et al., 1996; Naughton et al., 2000; Blier and Ward, 2003). However, all these antidepressants including SSRIs have a delayed onset of action, although the extracellular 5-HT level is increased several hours after drug intake. In addition, there are approximately one third of depressed patients that do not respond to these antidepressants, and enhancing 5-HT reuptake by the transporter enhancer tianeptine has a similar effect as inhibiting 5-HT reuptake by SSRIs in treating anxiety/depression (Lôo et al., 1992; Lacasse and Leo, 2005; Duman and Aghajanian, 2012). In this investigation, we used two kinds of central 5-HT-deficient mouse lines to explore the level of anxiety- and depression-like behaviors, and found that the anxiety-like behaviors were decreased but depression-like behaviors were similar to control mice (Figure 1). The depression-like behaviors in *Tph2*-deficient mice is still be a controversial topic: the depression-like behavior shown by tail-suspension test was not changed in *Tph2* knockout mice (Savelieva et al., 2008), while it was increased in *Tph2* knockin mice (Belmaker et al., 2008). In addition, it has been reported that the deletion of 5-HT1A receptor in mice increased anxiety, but reduced the immobility in the forced swimming test (Ramboz et al., 1998). Thus, opposite effects in anxiety- and depression-like behaviors by the manipulations of 5-HT system are not unique in our 5-HT-deficient mice. Although it may be argued that the forced swimming and tail suspension tests are behavioral indexes for screening chemicals having potential antidepressant effects in humans, they are also widely used to characterize the depression-like behaviors in rodents (Cryan et al., 2005). A recent finding demonstrates that ketamine, an antagonist of the NMDA receptor, produces rapid, robust and sustained antidepressant effects (Berton and Nestler, 2006; Duman and Aghajanian, 2012). Thus, other mechanisms such as the glutamate and adult hippocampal neurogenesis may play critical roles in depression patients resistant to SSRIs treatments. Nevertheless, our data demonstrate that 5-HT deficiency in the brain may not be sufficient to lead to depression- and anxiety-like behaviors in mice.

As mentioned above, a high prevalence of anxiety/depression is present in patients with diabetes mellitus (Ciechanowski et al., 2000; Anderson et al., 2001). After confirming the loss of Pet1-expressing cells in our DT-treated PC/DTR mice, we assessed them for symptoms of diabetes mellitus. As expected from the loss of islet cells, the mice exhibited several typical symptoms of diabetes mellitus, including polyphagia, polydipsia, polyuria and high blood glucose levels (Figure 3). The increased anxiety-like behaviors were observed in DT-treated PC/DTR mice (Figure 4), which is consistent with the con-occurrence of diabetes mellitus and anxiety in clinical observation. On the other hand, the expected increase of the level of depression-like behaviors was not observed. However, the hippocampal neurogenesis was significantly increased in our DT-treated PC/DTR mice (Figures 4, 5). In conventional *Tph2* knockout mice (Klempin et al., 2013) and PC/Lmx1b mice (unpublished observation), the adult hippocampal neurogenesis is not changed, but it is enhanced in diabetic db/db mice (Ramos-Rodriguez et al., 2014). Thus, it is likely the diabetic complication itself or together with the adult depletion of serotonergic neurons leads to the enhanced hippocampal neurogenesis in DT-treated PC/DTR mice. Previous studies have shown that the enhanced adult hippocampal neurogenesis played critical role in mediating SSRIs-induced antidepressant effect in mice (Malberg et al., 2000; Santarelli et al., 2003). Running wheel training enhances the adult hippocampal neurogenesis in rodents, and this may be a potential mechanism of the therapeutic effects by exercise on alleviating the symptoms of major depression as well as other behavioral defects (Ernstn et al., 2006; Lucassen et al., 2010; Wolf et al., 2011). It is possible that treatments (e.g., physical exercise), not limited to SSRIs, could promote adult hippocampal neurogenesis may be beneficial in alleviating depression symptoms.

Interestingly, the patients of wolfram syndrome often suffer from depressive states, and this disease is associated with the juvenile (non-autoimmune) diabetes. As the mouse model of wolfram syndrome, Wfs1-deficient mice displayed increased anxiety but normal depression level (Luuk et al., 2009). The immobility time of Wfs1-deficient mice in the forced swimming and tail suspension tests did not differ from that of wild-type littermates. However, there was augmented response to the administration of antidepressant drugs (imipramine and paroxetine) (Visnapuu et al., 2013). Similarly, our DT-treated PC/DTR mice also showed increased anxiety but a lower level of depression-like behaviors. Therefore, it may be a certain overlap of behavioral phenotypes between the Wfs1-deficient and our DT-treated PC/DTR mice. We supposed that 5-HT system may have some correlations with Wfs1 gene or wolfram syndrome.

Pet1 is restricted to the serotonergic neurons in the brain, but is not detected in non-serotonergic cells intermingled in the raphe nuclei of the brain stem (Hendricks et al., 1999, 2003). In addition, because wild type mice were used as control, some behavioral alterations observed in the PC/DTR and PC/Tph2 mice may be attributed to the possible subtle behavioral and neurobiological abnormalities that may exist in the *Pet1*-Cre mice, Rosa26-DTR mice and/or *Tph2*^flox/flox^ mice. In this study, we took advantage of the Pet1-driven Cre expression to deplete central serotonergic neurons, and this may not directly affect the survival of non-serotonergic neurons, but may affect their function as serotonergic neurons have functional connections with these neurons (Liu et al., 2002). On the other hand, it should be noted that the depletion of serotonergic neurons in DT-treated PC/DTR mice may lead to some degeneration-associated effects such as reactive gliosis, which may be implicated in behavioral alterations observed. Thus, although multiple mouse lines were used to address the relationship between the central 5-HT deficiency and anxiety- or depression-like behaviors, we could not exclude the possibility that the effects on these behavioral performances are caused indirectly by possible compensations and other unknown changes that are associated with the loss of 5-HT transmitter or serotonergic neurons.

## Conclusion

In summary, reduced anxiety-like but unchanged depression-like behaviors were observed in the two central 5-HT-deficient mice lacking either 5-HT itself or serotonergic neurons. We generated a novel mouse model in which the central serotonergic neurons and pancreatic islet cells were depleted in adulthood. These mice exhibited typical symptoms of diabetes mellitus, increased anxiety-like but a lower level of depression-like behaviors, and increased adult hippocampal neurogenesis. Our results suggest that 5-HT deficiency may not be sufficient to induce anxiety/depression-like behaviors in mice, and enhanced hippocampal neurogenesis may contribute to the absence of depression-like behaviors in central 5-HT-deficient mice with diabetes mellitus.

## Conflict of interest statement

The authors declare that the research was conducted in the absence of any commercial or financial relationships that could be construed as a potential conflict of interest.
